# Genomic and functional diversity of the human-derived isolates of *Faecalibacterium*

**DOI:** 10.3389/fmicb.2024.1379500

**Published:** 2024-05-30

**Authors:** Wenxi Li, Xiaoqian Lin, Hewei Liang, Zhinan Wu, Mengmeng Wang, Jingxi Sun, Xiaofang Li, Wenxin He, Xiaowei Gao, Tongyuan Hu, Liang Xiao, Yuanqiang Zou

**Affiliations:** ^1^BGI-Shenzhen, Shenzhen, China; ^2^School of Biology and Biological Engineering, South China University of Technology, Guangzhou, China; ^3^BGI Research, Wuhan, China; ^4^College of Life Sciences, University of Chinese Academy of Sciences, Beijing, China; ^5^BGI College and Henan Institute of Medical and Pharmaceutical Sciences, Zhengzhou University, Zhengzhou, China; ^6^Shenzhen Engineering Laboratory of Detection and Intervention of Human Intestinal Microbiome, BGI-Shenzhen, Shenzhen, China; ^7^Laboratory of Genomics and Molecular Biomedicine, Department of Biology, University of Copenhagen, Universitetsparken, Copenhagen, Denmark

**Keywords:** *Faecalibacterium*, gut microbiology, pan-genome, carbohydrate enzymes, probiotics

## Abstract

**Introduction:**

*Faecalibacterium* is one of the most abundant bacteria in the gut microbiota of healthy adults, highly regarded as a next-generation probiotic. However, the functions of *Faecalibacterium* genomes from cultured strains and the distribution of different species in populations may differ among different sources.

**Methods:**

We here performed an extensive analysis of pan-genomes, functions, and safety evaluation of 136 *Faecalibacterium* genomes collected from 10 countries.

**Results:**

The genomes are clustered into 11 clusters, with only five of them were characterized and validly nomenclated. Over 80% of the accessory genes and unique genes of *Faecalibacterium* are found with unknown function, which reflects the importance of expanding the collection of *Faecalibacterium* strains. All the genomes have the potential to produce acetic acid and butyric acid. Nine clusters of *Faecalibacterium* are found significantly enriched in the healthy individuals compared with patients with type II diabetes..

**Discussion:**

This study provides a comprehensive view of genomic characteristic and functions and of culturable *Faecalibacterium* bacterium from human gut, and enables clinical advances in the future.

## 1 Introduction

*Faecalibacterium*, belonging to Oscillospiraceae of Bacillota, is a genus of extremely oxygen-sensitive bacteria (Duncan et al., 2002). Based on the List of Prokaryotic names with Standing in Nomenclature (LPSN) [(Meier-Kolthoff et al., 2022), https://lpsn.dsmz.de/], only six species of this genus have been named validly, including *Faecalibacterium prausnitzii* (*F. prausnitzii*), *F. longum*, *F. butyricigenerans*, *F. duncaniae*, *F. gallinarum,* and *F. hattorii*. Evidence has proven that the colonization of *F. prausnitzii* usually happens during the late infancy (Laursen et al., 2017), and *F. prausnitzii* increases rapidly during the first year of lives (Bäckhed et al., 2015). A meta-analysis of 7,907 human guts showed that *F. prausnitzii* was detected in 85% of the samples, with the average abundance of 6.5% (De Filippis et al., 2020).

The decline in the abundance of *Faecalibacterium* is found closely related to gastrointestinal diseases and systemic diseases. Studies have shown that *Faecalibacterium* was significantly reduced in gut of patients with inflammatory bowel disease (IBD) (Frank et al., 2007), Crohn’s disease (Martinez-Medina et al., 2006), type 2 diabetes (Furet et al., 2010; Qin et al., 2012), obesity (Verdam et al., 2013), Parkinson’s disease (Li et al., 2017), and Alzheimer’s disease (Haran et al., 2019). *F. prausnitzii* can produce anti-inflammatory metabolites such as butyric acid and peptides (Zou et al., 2021). *F. prausnitzii* A2-165, which was classified as *F. duncaniae* as present (named *F. duncaniae* A2-165 below), was proven to have the ability to reduce the severity of inflammation and enhance the intestinal epithelial barrier (Sokol et al., 2008; Martín et al., 2014, 2015; Munukka et al., 2017), exerting an anti-asthmatic effect through short-chain fatty acid (SCFA) production (Hu et al., 2021). This evidence indicate that the presence and activity of *Faecalibacterium* species may be a biomarker of human health, making them suitable as next-generation probiotics.

Based on metagenomic approaches, biomarkers associated with diseases can be identified. However, these approaches often rely on read allocation or assembly binning based on reference databases, which may lack species-level matching and limit subsequent studies. Furthermore, the lack of cultured strains for most biomarkers restricts functional validation in metagenomic association analysis, which often only identifies potentially disease-associated bacterial species and potential functions without experimental validation. *Faecalibacterium* play an important role in human microbiota and also have a great impact on human health. However, the intervention experiments on diseases have more often been carried out with *F. duncaniae* A2-165 by now. Lack of *in vivo* and *in vitro* verification experiments of probiotic functions of other *Faecalibacterium* bacteria limits the in-depth research and applications of this probiotic genus.

Research studies about comparative genome analysis of *F. prausnitzii* has been published (Fitzgerald et al., 2018; Bai et al., 2022; Fabbrini et al., 2022), but current analysis of other species of *Faecalibacterium* (Benevides et al., 2017) are not enough. To further explore the genomic diversity and probiotic functions of *Faecalibacterium* to provide guidance for the selection of potential probiotics in disease intervention, we collected 136 *Faecalibacterium* genomes and performed an extensive exploration on them. In this study, we conducted a pan-genome analysis of *Faecalibacterium* genomes to explore genomic diversity and functional diversity and conducted analysis of probiotic function and safety analysis at the strain level. We also identify *Faecalibacterium* taxa that are significantly enriched in healthy people or patients with diseases. This study could be the basis of the clinical treatment and probiotics application of *Faecalibacterium* in the future.

## 2 Materials and methods

### 2.1 Collection of cultivated genomes of *Faecalibacterium* from human gut

This study collected 148 genomes of *Faecalibacterium* cultured from the human intestine on March 2022. Twenty-nine genomes of them were collected from the expanded Cultivated Genome Reference (CGR2) (Lin et al., 2023) and other genomes were downloaded from the Genbank of National Center for Biotechnology Information (NCBI, https://www.ncbi.nlm.nih.gov/). The genomes of “isolated” strains were manually selected. Genome quality was evaluated using CheckM (v1.1.2) (Parks et al., 2015), and the genomes with completeness over 95% and contamination less than 5% were retained. Finally, 136 genomes were retained for the further analysis.

### 2.2 Genomic characteristic statistic, phylogenetic, and taxonomic determination

The stat command of seqkit (v2.2.0) (Shen et al., 2016) was used to calculate genome size, and the fx2tab command was used to calculate G + C content. 16S rRNA gene sequences of the genomes were extracted by barrnap (v0.9).^1^ FastANI (v1.32) (Jain et al., 2018) was used to calculate the ANI value between genomes, and the R package hclust was used to perform species-level clustering of the generated ANI matrices. The threshold of 95% was used as the cutoff for bacterial species definition.

The “classify_wf” and “infer” modules of GTDB-Tk (v2.1.0) (Chaumeil et al., 2022) are used for species annotation and construction of genome phylogenetic trees, respectively. GTDB-Tk uses Prodigal to predict genes and HMMER to identify 120 marker genes of bacteria and then compares it with the marker genes in the bacterial reference phylogenetic tree of the Genome Database Taxonomy [GTDB, release207_v2 (Parks et al., 2022)]. The online tool iTOL (Letunic and Bork, 2021)^2^ was used for phylogenetic tree visualization and information annotation.

### 2.3 Calculation and functional analysis of pan-genomes

The protein-coding sequences (CDS) of each genome were predicted and annotated using Prokka v1.14.6 (Seemann, 2014). The amino acid sequences were mapped to gene families with the identity of 50% using the USEARCH tool of BPGA V1.3 (Chaudhari et al., 2016). A binary matrix showing the presence or absence of genes was used for iterative calculations of the pan-genome. Shared genes were calculated and plotted as a core curve, and all genes were calculated and plotted as a pan-genome curve, based on 200 times of iterative calculations. The gene families are classified into core genes, accessory genes, and unique genes. The “power3P” model from the R package ggtrendline was used to fit the pan-genome curve and core genome curve, and the “exp3P” model from the R package ggtrendline was used to estimate the parameter values.

Functional annotation was performed using eggNOG-mapper v2 (Cantalapiedra et al., 2021) [eggNOG database version 5.0.2 (Huerta-Cepas et al., 2019)]. The results of KEGG pathway classes were extracted from the eggNOG-mapper results.

### 2.4 Functional annotation of genomes

Mapping to the CAZy (Carbohydrate-Active enZYmes) database (Drula et al., 2022), the dbCAN3 (Zheng et al., 2023) was used for CAZymes annotation. dbCAN3 integrates two annotation methods and three databases, including HMMER search against the dbCAN CAZyme domain HMM database, DIAMOND search against the CAZy database, and HMMER search against CAZy subfamilies to infer their substrates. To ensure annotation accuracy, the results were compiled by summarizing the outputs of the three methods and removing those CAZymes found only by one method.

The pathway of short-chain fatty acid (SCFA) biosynthesis from pyruvate to acetate, butyrate, and propionate was referred to previous research studies (Bhatia and Yang, 2017; Louis and Flint, 2017). The amino acid sequences of these enzymes were retrieved and downloaded from the map00620 (pyruvate metabolism), map00650 (butanoate metabolism), and map00640 (propanoate metabolism) in the KEGG database (Kanehisa and Goto, 2000) (Kyoto Encyclopedia of Genes and Genomes, https://www.kegg.jp/). We used Blastp v2.2.26 (Camacho et al., 2009) to identify the gene-encoding enzymes related to SCFA biosynthesis in *Faecalibacterium* genomes with e-value = 0.01, identity >60%, and coverage >50%.

We used antiSMASH v6.0.0 (Blin et al., 2021) to predict microbial secondary metabolite biosynthetic gene clusters. The predicted gene cluster sequences were mapped to the (Kautsar et al., 2020) Minimum Information about a Biosynthetic Gene cluster (MIBiG) database, to find the most similar compounds. The parameters such as --cb-general, −-cb-knownclusters, and --cb-subclusters were used to blast the gene sequences to known classification of stimulated metabolism, and --smcogs was used to analyze the family of secondary metabolic genes.

### 2.5 Annotation of ARGs and VFs

We used the “main” feature with default parameter of Resistance Gene Identifier (RGI) version 5.2.0 to predict antibiotic resistance genes (ARGs) in each genome, mapping to the Comprehensive Antibiotic Resistance Database [CARD version 3.1.2 (Alcock et al., 2020)]. The genes predicted with the “strict” and “perfect” thresholds were selected for this study.

We then used Blastp v2.2.26 to identify the predicted virulence factors of each genome with the experimentally validated portion (setA) of the VFDB database (Liu et al., 2022) (Virulence Factor Database, http://www.mgc.ac.cn/VFs/). The gene occurrence was defined by the cutoff of e-value = 0.01, identity >60%, and coverage >50%.

### 2.6 Calculation of the distribution and abundance of *Faecalibacterium* species in metagenomes

To calculate the distribution and abundance of each cluster in different geographical locations among healthy populations, we downloaded 3,550 metagenomes of a Chinese cohort [part of the 4D-SZ (Jie et al., 2021)] from the CNGB Sequence Archive (CNSA) (Guo et al., 2020) of the China National GeneBank DataBase (CNGBdb) (Chen F. Z. et al., 2020) with accession number CNP0000426. Metagenomes from 8,244 healthy individuals from the Dutch cohort (Gacesa et al., 2022) were retrieved and downloaded from the European Genome-Phenome Archive (EGA) (Freeberg et al., 2022) with the accession number EGAS00001005027. Additionally, 661 metagenomes of healthy individuals from the Human Microbiome Project [HMP (Integrative HMP (iHMP) Research Network Consortium, 2019), https://portal.hmpdacc.org/] were downloaded.

To explore the association between *Faecalibacterium* and various diseases, we downloaded 995 metagenomes. In total, 171 metagenomes of healthy individuals and 214 metagenomes of patients with atherosclerotic cardiovascular disease in the ACVD cohort (Jie et al., 2017) were downloaded from the European Bioinformatics Institute (EBI) (Cantelli et al., 2022) with the accession number ERP023788. Overall, 104 metagenomes of healthy individuals and 152 metagenomes of patients with obesity were obtained from a precious study (Liu et al., 2017) and downloaded from the EBI database with the accession number PRJEB12123. A total of 183 metagenomes of healthy individuals and 171 metagenomes of patients with T2D were obtained from the T2D cohort (Qin et al., 2012) and downloaded from NCBI, with the accession number PRJNA422434.

Fastp (v0.23.1) was used to filter out low-quality reads and bases with partial parameters “--qualified_quality_phred 15 --complexity_threshold 30 --length_required 30”. Bowtie (v2.4.4) (Langmead and Salzberg, 2012) was used to remove host contamination by mapping the reads to the human genome (GRCh38). The “dereplicate” function of dRep (Olm et al., 2017) (URL: https://github.com/MrOlm/drep) was used to select the representative genome of each cluster. Eleven representative genomes were known as a bacterial genome reference in the Kraken2 (Wood et al., 2019) (v 2.1.2) database, and the combination of Kraken2 and Bracken (v2.6.1) (Lu et al., 2017) was used to estimate the abundance of representative genomes. Prevalence represented the percentage of the samples with abundance of *Faecalibacterium* over 0.1% in all samples.

### 2.7 Statistical analysis

Statistical tests were performed using R v4.1.2. For principal co-ordinates analysis (PCoA), Bray–Curtis dissimilarities were calculated using the vegdist function. The packages ggplot2 and pheatmap in R were used for plotting. Adobe Illustrator CC 2018 was used to adjust the colors and construct figures.

## 3 Results

### 3.1 Collection of *Faecalibacterium* genomes isolated from human gut

In our previous study, we constructed a large-scale collection of cultivated genome reference (CGR2) (Lin et al., 2023) of human gut including 29 genomes of *Faecalibacterium*. Moreover, we isolated two novel strains of *Faecalibacterium*, *F. longum* CM04-06 and *F. butyricigenerans* AF52-21 (Zou et al., 2021). To expand the knowledge of *Faecalibacterium* genomes, we further downloaded 107 genomes from the Genbank of National Center for Biotechnology Information (NCBI, https://www.ncbi.nlm.nih.gov/genbank/). Genomes were confirmed with >90% completeness and < 5% contamination, according to CheckM (Parks et al., 2015). In total, 136 genomes that isolated from human gut of 10 countries of Asia, Europe, North America, and Oceania were included in this study (Supplementary Table S1).

**Figure 1:**
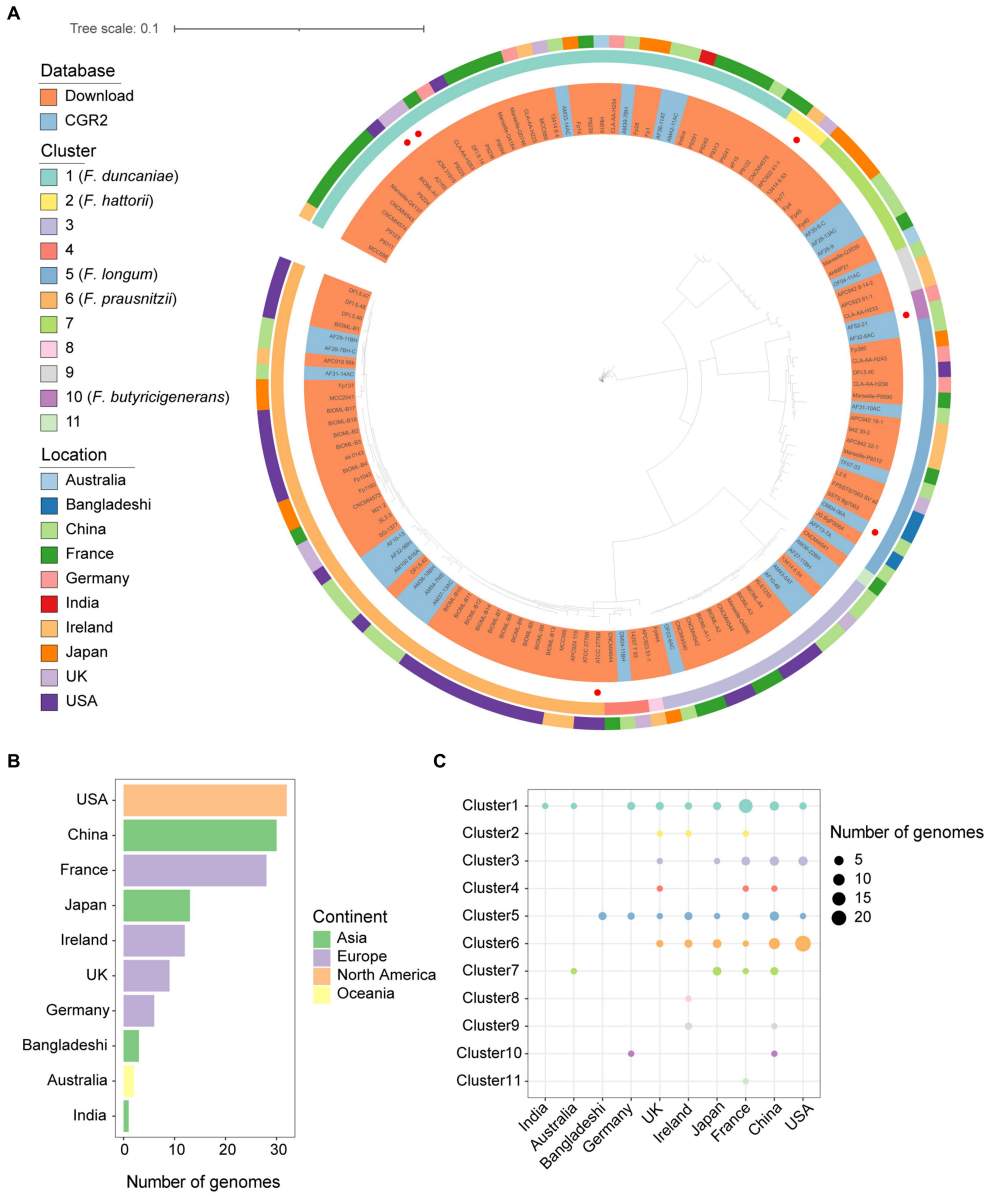
Genomic diversity of cultivated *Faecalibacterium* in human gut. **(A)** Phylogenetic tree of 136 cultivated genomes of *Faecalibacterium*. The strains were colored by the source of genomes. The type strains according to LPSN database were highlighted with a red point in the innermost circle. The second circle is colored according to the cluster. The third circle is colored according to the continents from which strains were isolated. **(B)** The bar plot shows the number of genomes collected from different countries and colored according to the continents. **(C)** The scatter plot shows the distribution of genomes among different clusters and countries.

With the cutoff of 95% average nucleotide identity (ANI), the genomes of *Faecalibacterium* were classified into 11 species-level clusters. Research (De Filippis et al., 2020) in 2020 showed that only eight clusters had representative cultivated genomes. We contributed three species-level clusters of *Faecalibacterium* that had not cultivated representatives before. Among the clusters, there are only five clusters characterized and validly nomenclated (Figure 1A), which are *F. duncaniae* (Cluster 1), *F. hattorii* (Cluster 2), *F. longum* (Cluster 5), *F. prausnitzii* (Cluster 6), and *F. butyricigenerans* (Cluster 10). Significant differences can be observed among the genome sizes, gene numbers, and GC contents of the 11 clusters of *Faecalibacterium*. Cluster 6 carried larger genome size and gene number, and the genomes of Cluster 1 had a wide range of genome size and gene number (Supplementary Figure S1A). According to the heatmap of the ANI values, the genomes were also classified into 11 clusters (Supplementary Figure S1B). These *Faecalibacterium* genomes were obtained from 10 countries. Moreover, the number of *Faecalibacterium* genomes obtained from the US were the most (*n* = 32), followed by China (*n* = 30) and France (*n* = 28) (Figure 1B). Most of the genomes (66.67%) isolated from the US were classified into Cluster 6. Over 50% of the genomes of Cluster 1 were obtained from France. Genomes of Cluster 1 could be obtained in all the countries except Bangladesh, indicating that it might widely exist in human gut. In total, 29 out of 30 of the *Faecalibacterium* genomes obtained from China were contributed by CGR2. Even though the genomes obtained from the US were the most, cultivated *Faecalibacterium* genomes of China and France were more diverse (Figure 1C), which indicated that the newly isolated genomes greatly expand the genetic diversity of *Faecalibacterium*. It was also found that the pairwise similarity among 16S rRNA gene sequences predicted from the whole genomes of all genomes were mostly higher than 97%, and the ANI values between clusters were all lower than 95% (Supplementary Figure S2). This result proved that 16S rRNA gene sequencing is not precious enough when identifying the taxonomy of *Faecalibacterium* strains, which limited the in-depth study of this genus.

### 3.2 Pan-genome of *Faecalibacterium* from human gut

**Figure 2:**
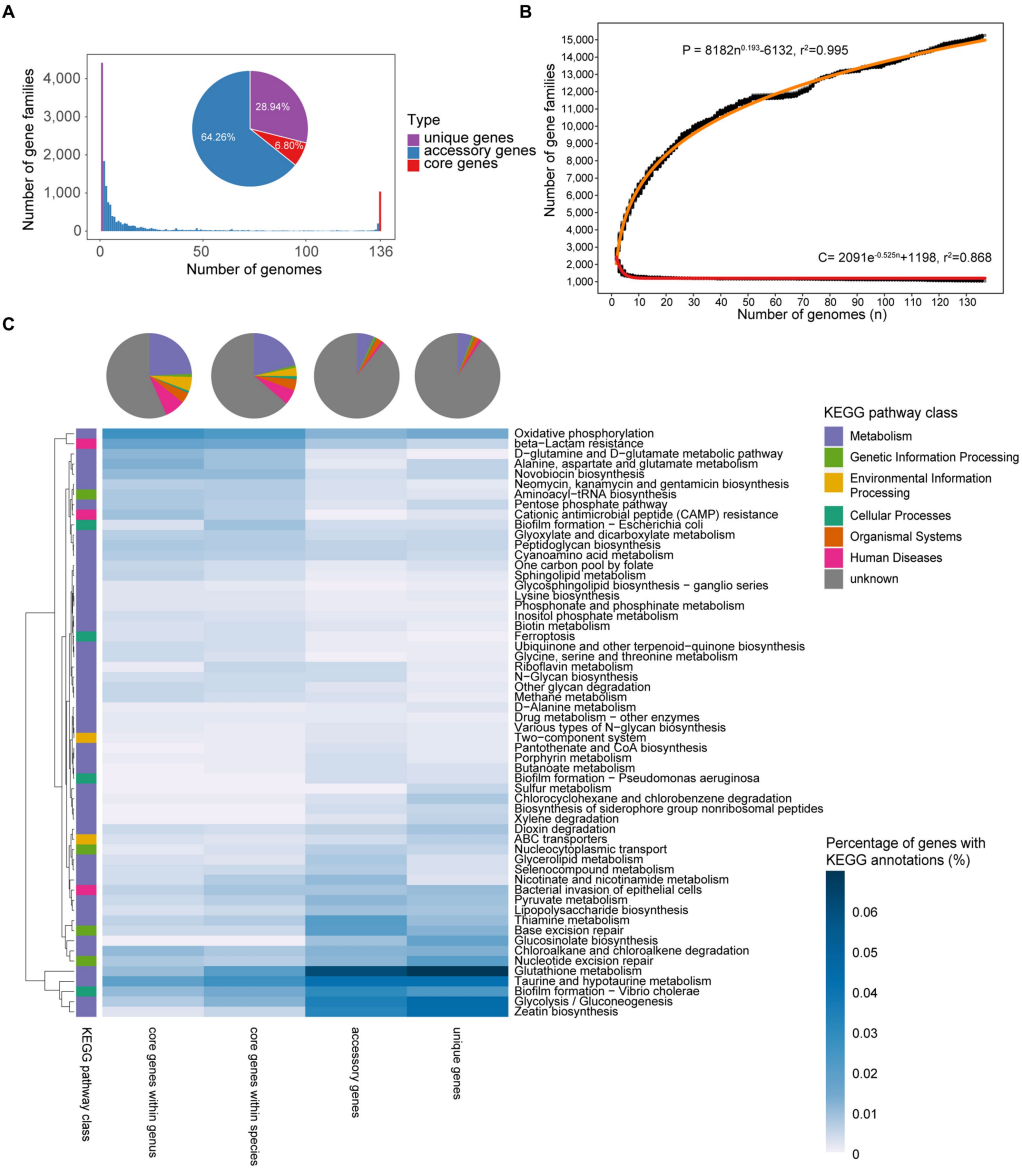
Pan-genome profile of *Faecalibacterium*. **(A)** Bar chart shows the number of gene families shared between different accumulated numbers of genomes. The pie plot shows the distribution of three types of genes in *Faecalibacterium* genomes. They are both colored according to the type of genes. **(B)** Fitting curves (red) of pan-genome and core genome. Black points represent the number of gene families carried by a random combination of corresponding number of genomes. The functional relationship equation between pan-genome size (P) and genome amount (*n*), and core genome size (C) and genome amount (*n*) are shown beside the curves. **(C)** Functional annotation and differential pathways of core genes, accessory genes, and unique genes. The heatmap shows the KEGG pathways differing among different types of genes, and the heat values in the heatmap represent the percentage of genes with KEGG annotation in all genes of corresponding type. The pie chart shows the distribution of functional categories among different types of genes.

A pan-genome consists of a core genome, accessory genes, and unique genes (Tettelin et al., 2005). In total, 381,241 protein-coding sequences (CDS) were predicted from 136 *Faecalibacterium* genomes. Based on the amino acid similarity of 95%, the CDSs were clustered into 15,261 non-redundant clusters of orthologous groups (which are gene families). Among all the gene families, 64.26% belonged to accessory genes and 28.94% belonged to unique genes. Only 1,038 gene families (6.80%) belonged to core genes, showing a high level of genetic diversity of these *Faecalibacterium* genomes (Figure 2A; Supplementary Table S2). Applying Heaps’ law (Tettelin et al., 2008), the pan-genome curve was predicted. Heaps’ alpha value was 0.193, which represented an open curve, indicating that the genetic diversity of this genus was still underestimated. With exponential function, the core genome curve was predicted and the core genome curve showed the opposite trend to the pan-genome curve. With the increase in the number of genomes, the core genome first decrease sharply and generally tends to be flat (Figure 2B).

To further find out the unique functions among different strains of *Faecalibacterium*, we predicted the pan-genome of each cluster. Similar to the result of ANI values, the phylogenetic tree based on core genes could still classified the genomes into 11 clusters, with 11 evolutionary branches. Moreover, the relationship of the phylogenetic relationship and the sources of genomes was not obvious (Supplementary Figure S3A). According to the pan-genome analysis of each cluster, the numbers of core genome increased by 543–1,584, showing that gene specificity may exist among different species. The genome OF04-11 AC from Cluster 9 had 441 unique genes, which is more than other genomes. Moreover, there were no unique genes existed in three genomes of *F. duncaniae*, three genomes of *F. prausnitzii*, and two genomes of Cluster 7. The fitting curve of pan-genomes of four clusters was all open, which indicated that a larger number of genomes were needed to identify more genes obtained by these species (Supplementary Figures S3B–E). It is necessary that we obtain more representative strains of *Faecalibacterium* by culture-based approach, to gain a deeper understanding of the genetic information of this genus.

The functions of core genes, accessory genes, and unique genes were predicted using Kyoto Encyclopedia of Genes and Genomes (KEGG) Orthology (KO) database (Kanehisa et al., 2021), with over 50% of them without any annotation. Notably, 90.46% of unique genes among each species and 89.16% of accessory genes among each species were unknown. This implies us that we could not know enough about most of the accessary and unique genes of these *Faecalibacterium* species at present, and these genes may make a significant contribution to the genetic diversity of *Faecalibacterium*. Among the annotated functions, genes related to metabolism were the most in core, accessory, and unique genes (Figure 2C). All the genomes harbored the genes related to D-glutamine and D-glutamate metabolic pathway, neomycin, kanamycin and gentamicin biosynthesis, sphingolipid metabolism, and other synthetic and metabolic pathways, which are necessary for maintaining the survival of microbes. However, genes related to zeatin biosynthesis, glycolysis/gluconeogenesis, taurine and hypotaurine metabolism, and thiamine metabolism were mostly accessory and unique genes. It indicated that these functions may only be shared among specific species or even strains, reflecting the functional differences among *Faecalibacterium* strains.

### 3.3 Functional variations of clusters of *Faecalibacterium*

**Figure 3:**
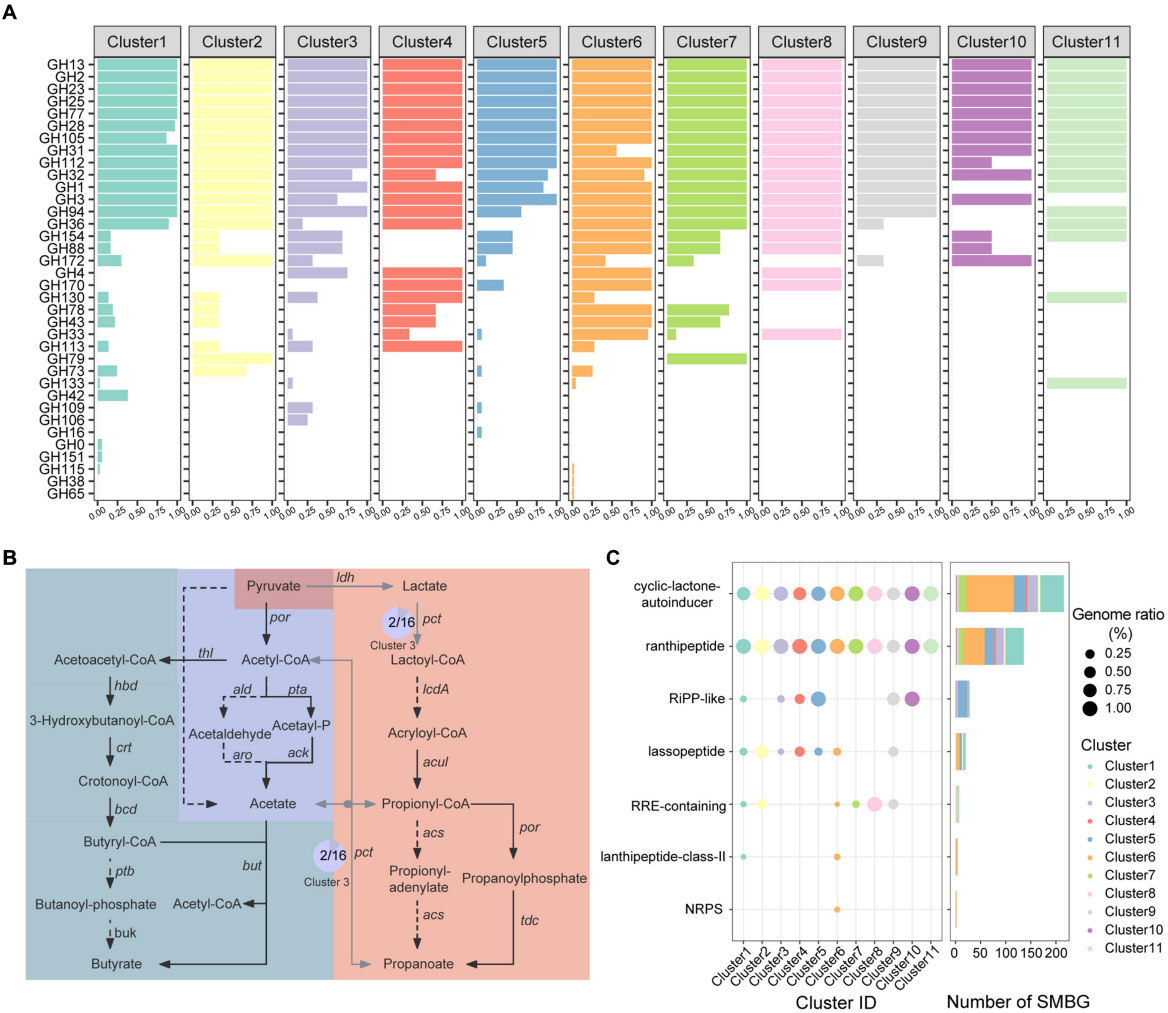
Functional annotation of cultivated *Faecalibacterium*. **(A)** The bar chart shows the CAZyme families harbored by 11 clusters of *Faecalibacterium*, colored according to the clusters. The abscissa represents the percentage of genomes which harbor corresponding CAZyme family in this cluster. **(B)** The pathway of short-chain fatty acid biosynthesis. The pathway with gray blue background is the biosynthesis of butyrate. The pathway in the middle with dark blue background is the biosynthesis of acetate. Moreover, the pathway on the right side with orange background is the biosynthesis of propanoate. Black solid line represents that the gene exist in all 136 genomes of *Faecalibacterium*. Gray solid line represents that the gene exist in part of genomes. Moreover, the dashed line represents that the gene is not harbored by any genome in this study. The pie charts show the proportion of the gene in the corresponding cluster, and the number in the pie chart represents the number of genomes harboring this gene. **(C)** The distribution of BGC in different clusters, which is colored according to the clusters. The point size in the scatter plot represents the percentage of the genomes harboring corresponding BGC in this cluster.

We performed an in-depth analysis on the functions of carbohydrate enzymes (CAZymes), short-chain fatty acid synthesis, and secondary metabolite synthesis among the *Faecalibacterium* genomes. Substantial percentages of the genomes of gut microbiome are responsible for carbohydrate degradation and nutrient absorption (Wardman et al., 2022). In total, 80 CAZyme families were annotated from 136 *Faecalibacterium* genomes. Genomes of Cluster 7 harbored the most of CAZymes, while genomes of Cluster 5 (*F. longum*), Cluster 9, Cluster 10 (*F. butyricigenerans*), and Cluster 11 harbored less amount of CAZymes (Supplementary Figure S4A; Supplementary Table S3A). Moreover, the significant difference was observed among the distribution of CAZymes of each cluster (*p* < 0.001) (Supplementary Figure S4B), indicating that the amount and composition were both different among each cluster of *Faecalibacterium*. Common glycoside hydrolase (GH) including GH2 (β-galactosidase), GH13 (α-amylase), GH23 (peptidoglycan lyase), GH25 (lysozyme), GH77 (amylomaltase) (Figure 3A), and glycosyl transferase (GT) including GT2 (cellulose synthase), GT4 (sucrose synthase), GT28 (1,2-diacylglycerol 3-β-galactosyltransferase), GT35 (glycogen or starch phosphorylase), and GT51 (murein polymerase) were harbored by all 136 genomes. Notably, CAZyme families of CE4 (acetyl xylan esterase) and CE9 (N-acetylglucosamine 6-phosphate deacetylase), which had been proven crucial for the amino sugar metabolism and peptidoglycan cell wall circulation in bacteria (Park, 2001), were also harbored by all 136 genomes of *Faecalibacterium*. Genomes of *F. prausnitzii* (Cluster 6) harbored significantly more genes encoding GH43 (β-xylosidase), GH78 (α-L-rhamnosidase), GH4 (maltose-6-phosphate glucosidase), GH170 (6-phospho-N-acetylmuramidase), and GH33 (sialidase or neuraminidase) but less genes encoding GH31 (α-glucosidase) (Figure 3A; Supplementary Figure S4C). To access mucin glycans, intestinal microbes must express the GH33 sialidases (also known as neuraminidases) (Glover et al., 2022). Among *Faecalibacterium* 136 genomes, 41 of 43 genomes of Cluster 6 (*F. prausnitzii*) harbored the gene encoding GH33. Moreover, one genome each for Cluster 3, Cluster 4, Cluster 5, Cluster 7, and Cluster 8 also harbored the gene encoding GH33. These results reflected that *Faecalibacterium* could utilize dietary- and host-derived carbohydrates, and *F. prausnitzii* had the ability to additionally degrade rhamnose and sialic acid. The difference in the composition of CAZymes may be related to different levels of ability of colonization in the human gut.

Short chain fatty acids (SCFAs) such as acetic acid, propionic acid, and butyric acid provide energy for intestinal epithelial cells and regulate the host’s immune system, which are important to maintain the human health (Martin-Gallausiaux et al., 2021). According to the previous articles (Bhatia and Yang, 2017; Louis and Flint, 2017), we mapped a metabolic pathway from pyruvate to these three types of SCFAs (Figure 3B). We then blasted the CDS predicted from 136 genomes with the amino acid sequences in this pathway. All the 136 genomes covered the complete pathway of acetic acid and butyric acid production, suggesting that the production of acetic acid and butyric acid might be a conservative function of this genus (Supplementary Table S3B). All genomes could catalyze acetyl-coenzyme A (Acetyl-CoA) to produce acetyl-phosphate (Acetyl-P) by phosphoacetyltransferase *pta* and finally produce acetic acid by acetic kinase *ack*. Additionally, two genomes of Cluster 3 (AM43-5AT and CNCMI14540) have the potential to generate acetic acid by one-step reaction. None of the genome covered the whole pathway of propanoate production. Only 9 genomes of Cluster 7 (AHM21, Fp4, Fp40, Fp45, Fp77, Marseille-Q3530, AF26-9, AF28-13 AC, and AF35-6-C) and 1 genome of Cluster 6 (MCC585) harbored the gene *ldh*. Moreover, only two genomes (AM43-5AT and CNCMI14540) of Cluster 3 harbored the gene *pct*. However, all genomes lacked the gene *lcdA*, which is not able to synthesize acryl coenzyme A (Acryloyl-CoA) from lactoyl coenzyme A (Lactoyl-CoA).

Some clusters of *Faecalibacterium* were found harboring the gene *ldhA* or *ldh*. The *ldhA* gene participates in the conversion of pyruvate to D-lactate (Bunch et al., 1997) while the *ldh* gene helps L-lactate production. All genomes of Cluster 3, Cluster 4, Cluster 8, Cluster 11, and Cluster 7 genomes *F. prausnitzii* harbored the *ldhA* gene. All the genomes of Cluster 7 and Cluster 1 of *F. prausnitzii* harbored the *ldh* gene. In addition, we found that some genomes of *Faecalibacterium* harbored the *cbh* gene. This gene encodes bile salt hydrolase (BSH), which is of great significance for lowering cholesterol and preventing cardiovascular diseases (Jones et al., 2013). Seven genomes of *F. duncaniae*, one genome of F. longum, six genomes of *F. prausnitzii*, and one genome of Cluster 7 harbored this gene. These results showed that some *Faecalibacterium* genomes harbored the probiotic functions which might related to the human health (Supplementary Figure S5).

We then used antiSMASH (V6.0.0) to explore the potential secondary metabolite biosynthetic gene clusters (SMBGs) in 136 genomes which were annotated. In total, 407 SMBGs of 7 types were identified from the genomes (Figure 3C). Cyclic lactone autoinducers are the most abundant type of SMBG, with 216 cyclic lactone autoinducers annotated in 119 genomes (Supplementary Table S3C). Autoinducers serve as signaling molecules involved in bacterial quorum sensing (Mukherjee and Bassler, 2019), allowing communication within and between different species. Except for a genome of Cluster 9 (OF04-11 AC), at least one radical non-α-carbon thioether peptide (Ranthipeptide) SMBG was annotated in all genomes. Ranthipeptides, previously known as “SCIFF peptides,” are ribosomally synthesized and post-translationally modified peptides (RiPPs) (Arnison et al., 2013). Recently, these peptides were proven having the potential to participate in quorum sensing mechanisms and played an important role in the regulation of microbiome composition (Chen Y. et al., 2020). In addition, SMBGs of RiPP-like peptides, lassopeptides, RiPP recognition elements (RRE-containing), lanthipeptides, and non-ribosomal peptide synthetases (NRPS) were harbored by the *Faecalibacterium* genomes. Some of these secondary metabolites have been reported to harbor antibacterial activity (Zhang et al., 2014; Hammami et al., 2015; Repka et al., 2017). It indicated that the strains of *Faecalibacterium* might participate in the interaction of microbial communities through the synthesis of bioactive substances. The antimicrobial activity associated with SMBGs of these genomes might contribute to the colonization of different ecological niches and the inhibition of specific pathogenic bacteria. In addition, *Faecalibacterium* has great potential for the discovery of new secondary metabolites.

### 3.4 Safety evaluation of cultivated genomes of *Faecalibacterium* in human gut

Similar to other probiotics, *Faecalibacterium* may harbor different antibiotic resistance genes or virulence factor-related genes, which could transfer between strains through gene exchange (Machado et al., 2022). It is necessary to perform a safety assessment before using a candidate probiotic for clinical intervention. We here analyzed the antibiotic resistance genes (ARGs) and genes encoding virulent factors (VFs) among the 136 genomes of *Faecalibacterium*.

In total, 20 types of antibiotic resistance genes were annotated from 136 *Faecalibacterium* genomes, with 5 of them being multidrug-resistant genes. These 20 ARGs, which were resistant to 16 types of antibiotics, were only harbored by a few genomes (Supplementary Figure S6; Supplementary Table S4A). Species or geographic specificity of ARGs among *Faecalibacterium* could not be observed in this result. According to the World Health Organization (2019), 19 out of 20 antibiotics are classified as critically important or highly important, except chloramphenicol. Additionally, 55 strains could not be annotated with any antibiotic resistance genes. It indicated that these 55 strains might be safer for clinical intervention than other strains harboring more ARGs.

A total of 24 virulence genes were annotated among all 136 *Faecalibacterium* genomes, encoding 14 types of virulence factors (Supplementary Figure S6; Supplementary Table S4B). Capsule, lipopolysaccharide (LPS), and molecular chaperone GroEL were the most abundant VFs that harbored by these genomes, which were ranked in a descending order of gene copy number. However, these annotated VFs are involved in bacterial immune regulation, stress survival, adhesion, and effector delivery systems, which are important for probiotics to colonize and compete with other bacteria. The absence of toxin-related virulence factor genes annotated indicated that these 136 *Faecalibacterium* genomes might not be threatening to human health. Therefore, these genomes might be safe for probiotic application and have the potential to be utilized in clinical treatment.

### 3.5 Distribution of cultivated genomes of *Faecalibacterium* from different populations

**Figure 4:**
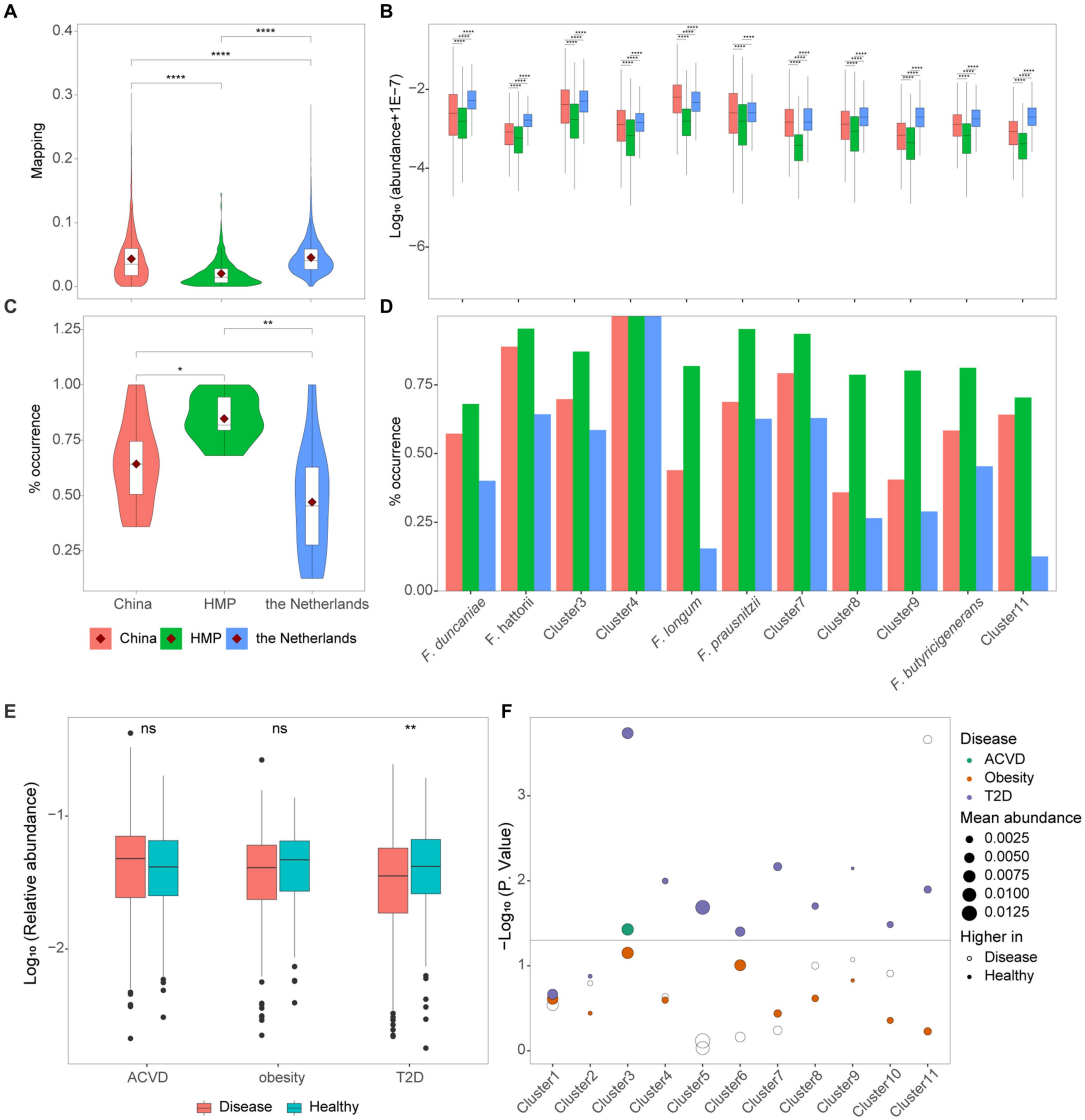
Distribution of cultivated *Faecalibacterium* in metagenomes. **(A)** The reads mapping to *Faecalibacterium* in three cohorts of healthy individuals. **(B)** The logarithm (base 10) of the abundance of 11 clusters of *Faecalibacterium* in the metagenomes of three different cohorts. **(C)** The prevalence of *Faecalibacterium* in the metagenomes of three different cohorts. The prevalence represents the percentage of samples with the abundance of each cluster higher than 0.1%. **(D)** The prevalence of 11 clusters of *Faecalibacterium* in three cohorts. Panels **(A–D)** are colored according to the cohort. * Represents *p* < 0.05, ** represents *p* < 0.01, *** represents *p* < 0.001, and **** represents *p* < 0.0001. **(E)** The abundance of *Faecalibacterium* genus in three case–control cohorts. **(F)** The scatter plot shows the difference of 11 *Faecalibacterium* clusters among 3 types of case–control cohorts, which are colored according to different diseases. The dashed line represents *p*-value = 0.05. The size of point represents the average abundance of each cluster in this cohort. The hollow point represents that higher abundance is observed in the disease group, while the solid point represents that higher abundance is observed in the healthy group.

To explore the distribution of *Faecalibacterium* in human gut among different cohorts, we calculated the relative abundance and prevalence of 11 clusters in three populations of healthy individuals from China, HMP, and the Netherlands. The average relative abundances of each cohort were 4.33, 2.04, and 4.54%, respectively (Figure 4A). Moreover, significant differences could be observed among these three populations. The relative abundances of 10 out of 11 clusters were significantly different among the cohorts of China, HMP, and the Netherlands (Figure 4B). *F. longum* was significantly enriched in the healthy population of China, while the other nine clusters were significantly enriched in the healthy population of the Netherlands. Moreover, for *F. prausnitzii*, the relative abundance in people participating in HMP was the significantly lowest, while the relative abundance among people from China and the Netherlands has no significant difference. The percentage of samples with the relative abundance of *Faecalibacterium* higher than 0.1% was considered as the prevalence. Even though the relative abundance of *Faecalibacterium* was found significantly the lowest in HMP cohort, the prevalence in the HMP cohort was significantly higher than the Chinese and Dutch cohorts (Figure 4C). The high abundance and low prevalence of *Faecalibacterium* in the Dutch cohort suggested that it might be present at high level of abundance in specific samples. Additionally, Cluster 4 was found in all the samples of the Chinese, HMP, and Dutch cohorts (Figure 4D).

Previous studies have found that the relative abundance of *Faecalibacterium* significantly reduced in the gut of patients with atherosclerotic cardiovascular disease (ACVD) (Jie et al., 2017; van den Munckhof et al., 2018), obesity (Liu et al., 2017; Maioli et al., 2021), and type 2 diabetes (T2D) (Qin et al., 2012; Gurung et al., 2020) and might play an important role in the intervention of the treatment of these diseases. To explore which strains of culturable *Faecalibacterium* in this collection that can be used for clinical research, we mapped 11 clusters to 995 metagenomes to identify potential associations with healthy control and disease. Metagenomic data of atherosclerotic cardiovascular disease (ACVD) (Jie et al., 2017), obesity (Liu et al., 2017), and type 2 diabetes (T2D) (Qin et al., 2012) were downloaded to investigate the relative abundance of the 11 clusters.

At the genus level, the relative abundance of *Faecalibacterium* was significantly lower in patients with T2D compared with the healthy control (*p* < 0.05). However, significant differences in the relative abundance of *Faecalibacterium* were not observed between people in healthy group and patients with ACVD and people in obesity group (Figure 4E). This result differs slightly from the original research, which may be due to a bias caused by an insufficient previous understanding of the taxonomy of the genus *Faecalibacterium*. Then, we explore differences at the species level. Compared with the patients suffering from T2D, Cluster 3, Cluster 4, *F. longum*, *F. prausnitzii*, Cluster 7, Cluster 8, Cluster 9, *F. butyricigenerans*, and Cluster 11 were significantly reduced in healthy people (Figure 4F). It indicated that these species are worthy of further investigation and have the potential to be applied in clinical invention of T2D, though many of them have not been characterized yet. For the ACVD, Cluster 3 was enriched in the healthy group, while Cluster 11 was enriched in the disease group. No significant difference in the relative abundance of each cluster was found in the obesity cohort.

## 4 Discussion

*Faecalibacterium* is one of the high-abundance taxa in the healthy human gut (De Filippis et al., 2020) and has the potential to be a new generation of probiotics. Only 5 out of 11 clusters have been characterized and taxonomically named so far, indicating that much of the taxonomy of *Faecalibacterium* remain unknown.

*Faecalibacterium* has an open pan-genome and a relatively small core genome, suggesting high genetic variability within this species. Over 80% of the accessory genes and unique genes were found with unknown function based on the databases up to now, and their contributions to the colonization, growth, and transmission of *Faecalibacterium* remain unknown.

Annotation of carbohydrate enzymes revealed differences in the potential utilization of carbohydrates among different clusters. Cluster 7 was found harbored the most diverse CAZyme families among the 11 clusters of *Faecalibacterium*. Our research emphasizes the potential of *Faecalibacterium* in synthesizing acetic acid and butyric acid, and its benign nature is due to the minimal presence of antibiotic resistance genes and virulence factors. These findings highlight the promise of the genus as a potentially safe probiotic candidate. One of the desired properties of probiotics is the ability to compete with pathogens. The exploration of SMBGs suggested that specific strains had the potential to produce antibiotics, which might contribute to their occupation of important ecological niches in the intestine or the competition with other bacteria. The analysis in this study shows different composition patterns of *Faecalibacterium* in different healthy populations of Chinese, Netherlands, and the HMP cohort, with *F. longum* being the predominant species in the Chinese healthy population. The *Faecalibacterium* species with significantly different abundance between healthy individuals and patients may represent potentially beneficial bacteria that can prevent or treat specific diseases. In total, 9 out of 11 *Faecalibacterium* clusters were enriched in the healthy group of type II diabetes cohorts, which might provide theoretical support for selecting suitable *Faecalibacterium* strains for subsequent *in vivo* and *in vitro* functional studies and disease interventions.

Butyrate has become an attractive target for type II diabetes (Arora and Tremaroli, 2021). It is also one of the fermentation products of *F. longum*, *F. prausnitzii*, and *F. butyricigenerans*. It was proven that the direct supplementation of butyrate salts and its derivatives have beneficial effects on the treatment of T2D (Mollica et al., 2017). However, using oral butyrate supplementation was not very effective according to the results of human trials (Bouter et al., 2018; Khosravi et al., 2022), possibly due to the short half-life of butyrate salts and the inability of conventional administration routes to simulate the sustained release and absorption of butyrate salts in the colon and circulation (Gill et al., 2018). Butyrate-producing bacteria offer an alternative approach for the treatment of T2D. Our study identified the pathway of the butyrate synthesis of *Faecalibacterium*. Due to the high butyrate production and higher abundance in human gut compared with other species of *Faecalibacterium*, *F. longum* CM04-06^T^ in this study (Zou et al., 2021) might have the potential to be an intervention strain for T2D. This genome only harbored one resistance gene AAC(6′)-Ie-APH(2″)-Ia, which is associated with aminoglycoside antibiotic inactivation. However, its biological function and intervention effects still need to be validated in the further experiment in animal models in the future.

However, there are still limitations of our work that not enough reference genomes of Faecalibacterium were included in this study. This might lead to an insufficient understanding of the taxonomic and functional information of Faecalibacterium up to now. Most of the *Faecalibacterium* genomes in this article were obtained from the United States, China, and France, with fewer genomes from other countries. This also leads to uneven distribution of the number of *Faecalibacterium* species and an incomplete understanding of the functional diversity of the genus. We call for extensive isolation of gut microbes from various countries to explore the functional diversity of *Faecalibacterium* more fully in different regions. In conclusion, we envisage that our study will serve as a useful summary of the characteristics and functions of genomes of cultivated *Faecalibacterium* in the human gut and a reference for the clinical application of *Faecalibacterium* in the future.

## Data availability statement

The original contributions presented in the study are included in the article/Supplementary material, further inquiries can be directed to the corresponding author. The data that support the findings of this study can be accessed in the zenodo with https://doi.org/10.5281/zenodo.10516060.

## Author contributions

WL: Data curation, Formal analysis, Investigation, Writing – original draft, Writing – review & editing. XqL: Conceptualization, Formal analysis, Investigation, Methodology, Validation, Visualization, Writing – original draft. HL: Formal analysis, Investigation, Methodology, Software, Writing – original draft. ZW: Formal analysis, Investigation, Visualization, Writing – original draft. MW: Formal analysis, Writing – original draft. JS: Methodology, Writing – original draft. XfL: Formal analysis, Writing – original draft. WH: Visualization, Writing – original draft. XG: Validation, Writing – review & editing. TH: Software, Writing – original draft. LX: Project administration, Writing – review & editing. YZ: Funding acquisition, Project administration, Supervision, Writing – review & editing.

## Footnotes

^1^ https://github.com/tseemann/barrnap

^2^ https://itol.embl.de/
