# Supplementary material for: Genomic and functional diversity of the human-derived isolates of Faecalibacterium
Source: Front Microbiol. 2024 May 30;15:1379500. doi: 10.3389/fmicb.2024.1379500 (PMC11169845; doi:10.3389/fmicb.2024.1379500)
Supplement: Supplementary file 1 [file Data_Sheet_1.ZIP › Supplementary_Material.docx]

Supplementary Material

Genomic and functional diversity of the human-derived isolates of Faecalibacterium

The Supporting Information contains:

**Figure S1.** Genomic characteristic of 136 genomes of cultivated *Faecalibacterium*.

**Figure S2.** The ANI values and 16S rRNA gene similarity within and between clusters of *Faecalibacterium*.

**Figure S3.** The pan-genes of *Faecalibacterium* genomes.

**Figure S4.** The CAZymes profile of *Faecalibacterium*.

**Figure S5.** The safety evaluation profile of *Faecalibacterium*.

**Table S1.** Genomic information of 136 genomes of *Faecalibacterium*.

This table contains the source, country, species, genome characteristics and other information of 136 *Faecalibacterium* genomes.

**Table S2.** Pan-genes profile of *Faecalibacterium*.

This table includes the number of genes in each pan-genome category obtained from 136 *Faecalibacterium* genomes

**Table S3.** The functional profile of genomes of *Faecalibacterium*.

This table includes the functional information of the *Faecalibacterium* genome, including (**Table S3A**) the carriage of carbohydrate-active enzymes (CAZymes), (**Table S3B**) the carriage of corresponding enzymes of short-chain fatty acids (SCFA) pathway, (**Table S3C**) the carriage of biosynthetic gene clusters (BGC).

**Table S4.** The safety evaluation of *Faecalibacterium* genomes.

This table includes the safety functions of the *Faecalibacterium* genome, including (**Table S4A**) antibiotics resistance genes (ARGs) and (**Table S4B**) virulence factors (VF).

The supplementary tables were additionally attached as excel files.


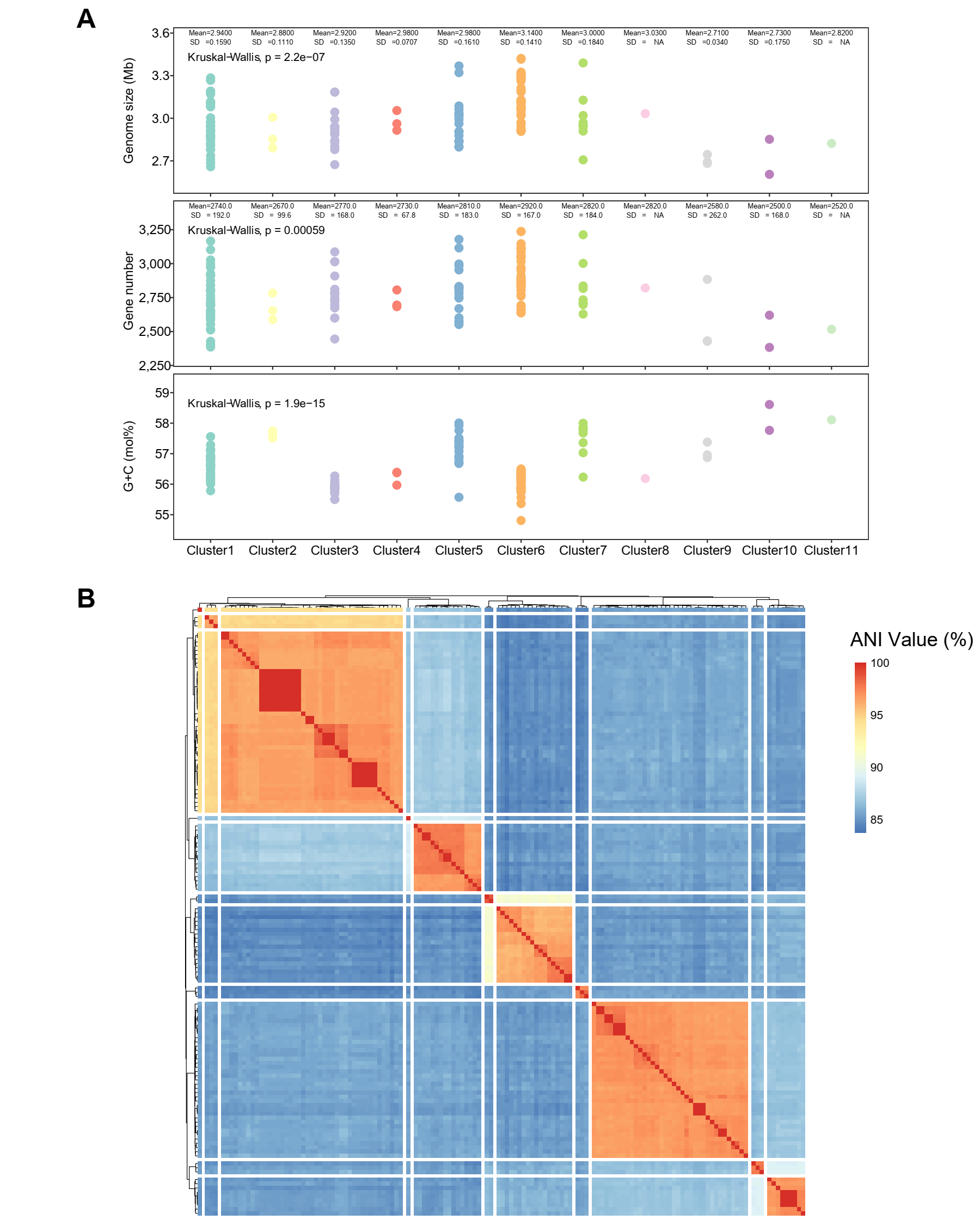


**Figure S1. Genomic characteristic of 136 genomes of cultivated *Faecalibacterium*. A**, The genome size, gene number, and the GC content of genomes in 11 clusters. The average number (mean) and standard deviation (SD) are shown above the points. Kruskal-Wallis test is used for overall difference test and the p-values are shown in the figure. **B**, The ANI matrix of 136 *Faecalibacterium* genomes*.* The order of genomes from left to right corresponds to the order of genomes from top to bottom.


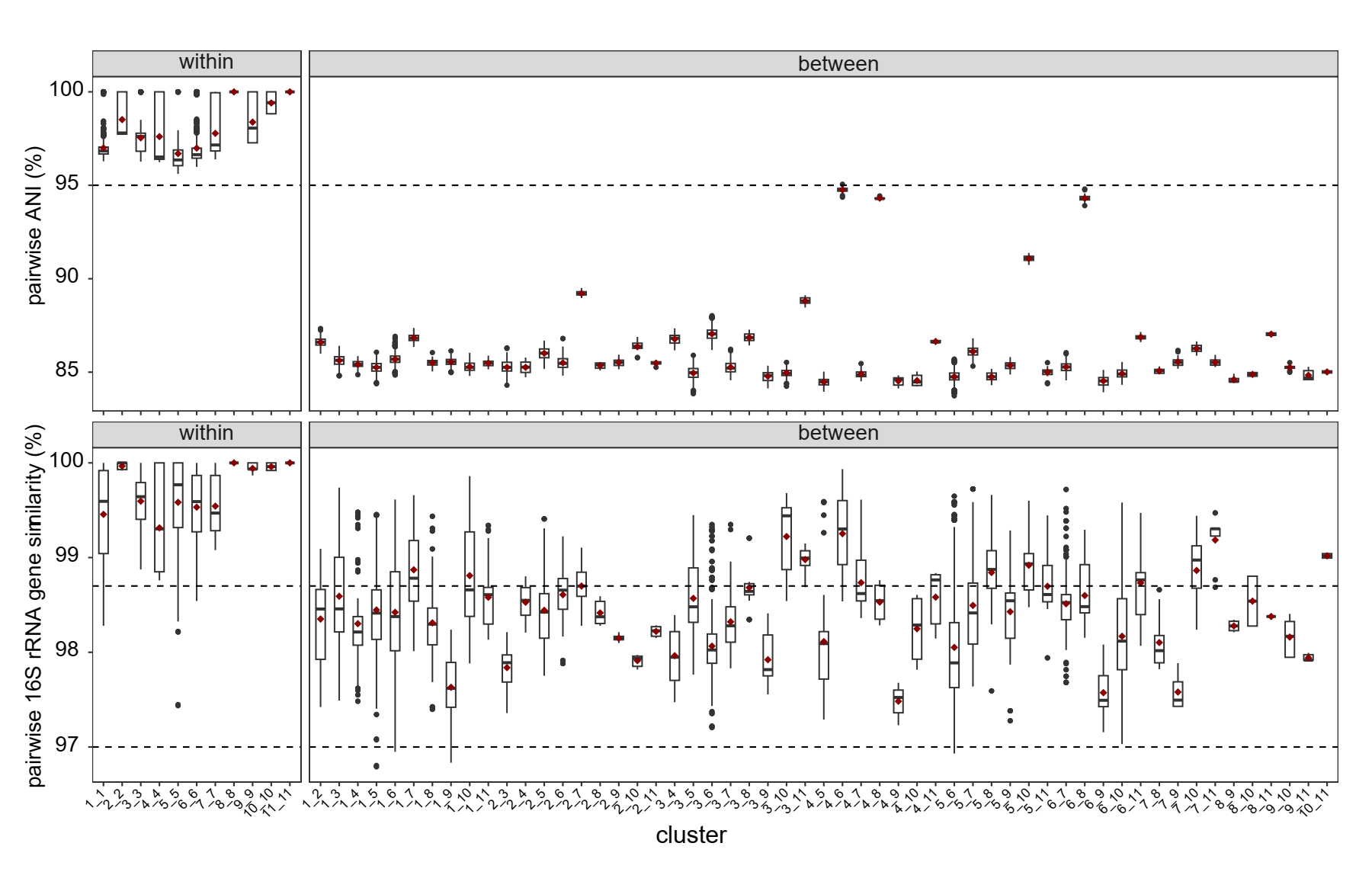
 **Figure S2. The ANI values and 16S rRNA gene similarity within and between clusters of *Faecalibacterium*.** The abscissa represents different combinations of clusters, and the points in the boxplot represent the similarity values between randomly selected genomes within each cluster.


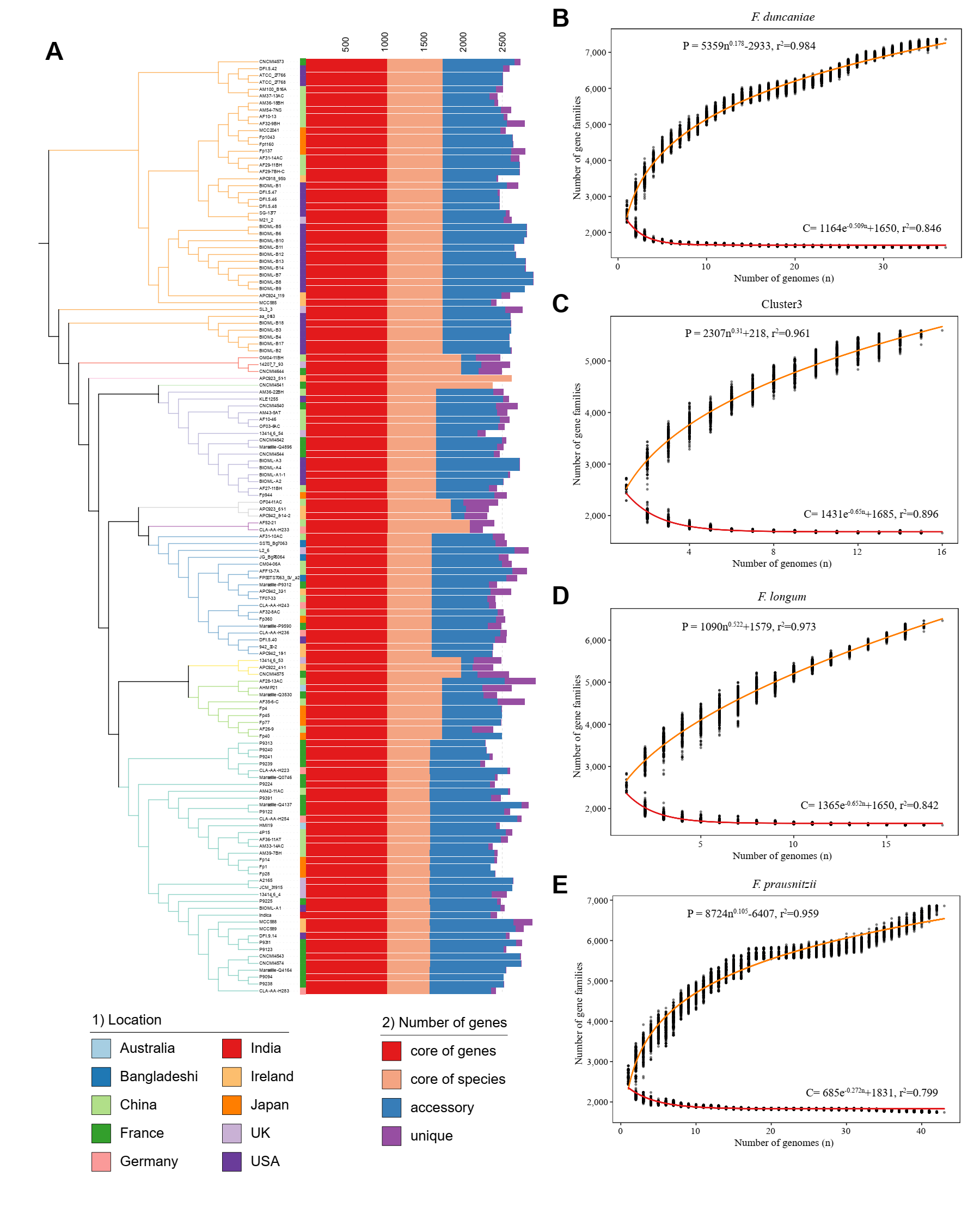
 **Figure S3. The pan-genes of *Faecalibacterium* genomes. A**, The phylogenetic tree is annotated with locations of the genomes and number of core genes among genus, core genes among species, accessory genes and unique genes. The branches are colored according to the clusters of genomes. **B-E**, The fitting curves of pan genome and core genome of four clusters with more genomes than other clusters, including *F. duncaniae* (**B**), Cluster 3 (**C**), *F. longum* (**D**), and *F. prausnitzii* (**E**).


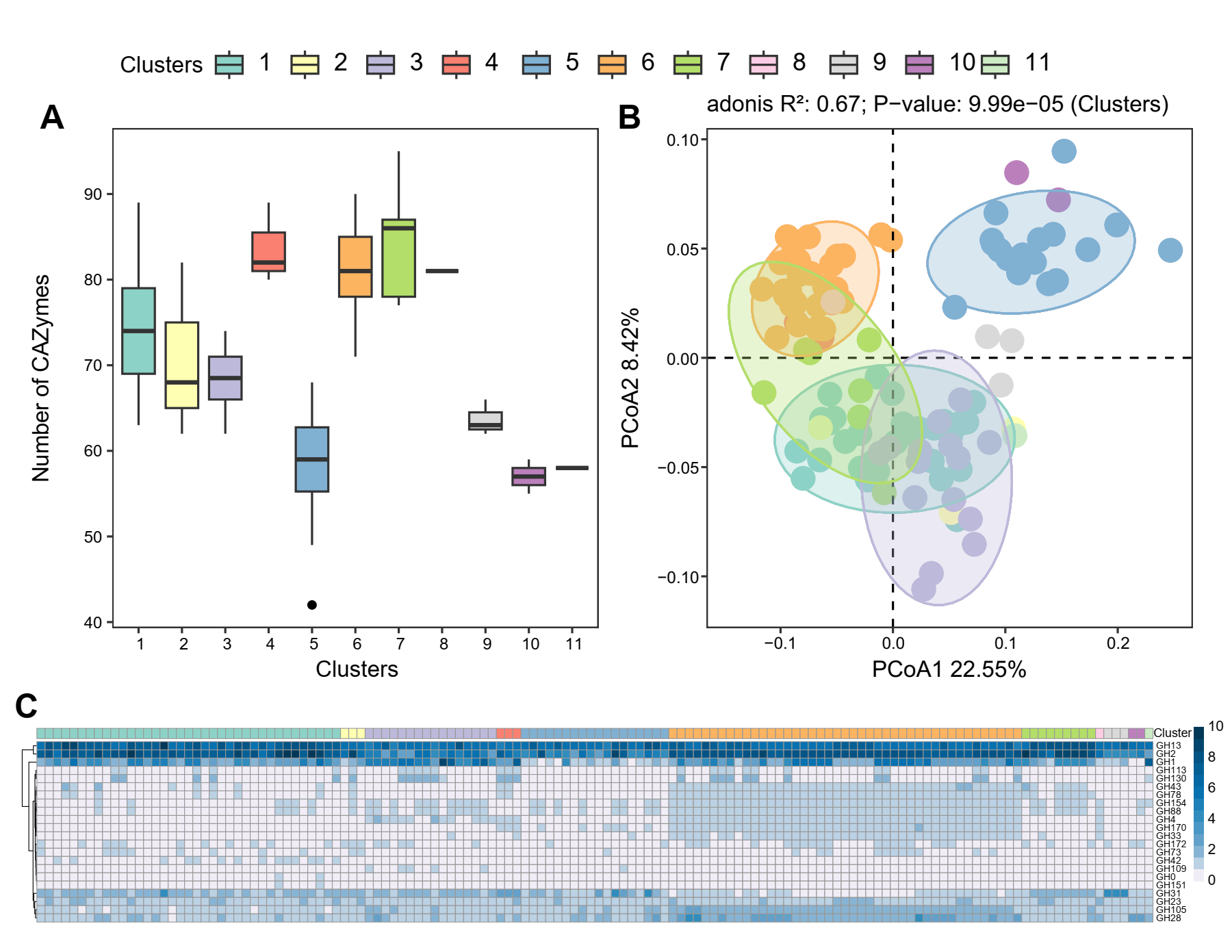
 **Figure S4. The CAZymes profile of *Faecalibacterium*. A**, The boxplot shows the number of CAZymes harbored by genomes of each cluster. **B**, The principal co-ordinates analysis (PCoA) of CAZymes shows the distance between clusters. Both panel a and b are colored according to the clusters. **C**, The heatmap of CAZymes shows the number of CAZymes harbored by genomes, colored according to the clusters.


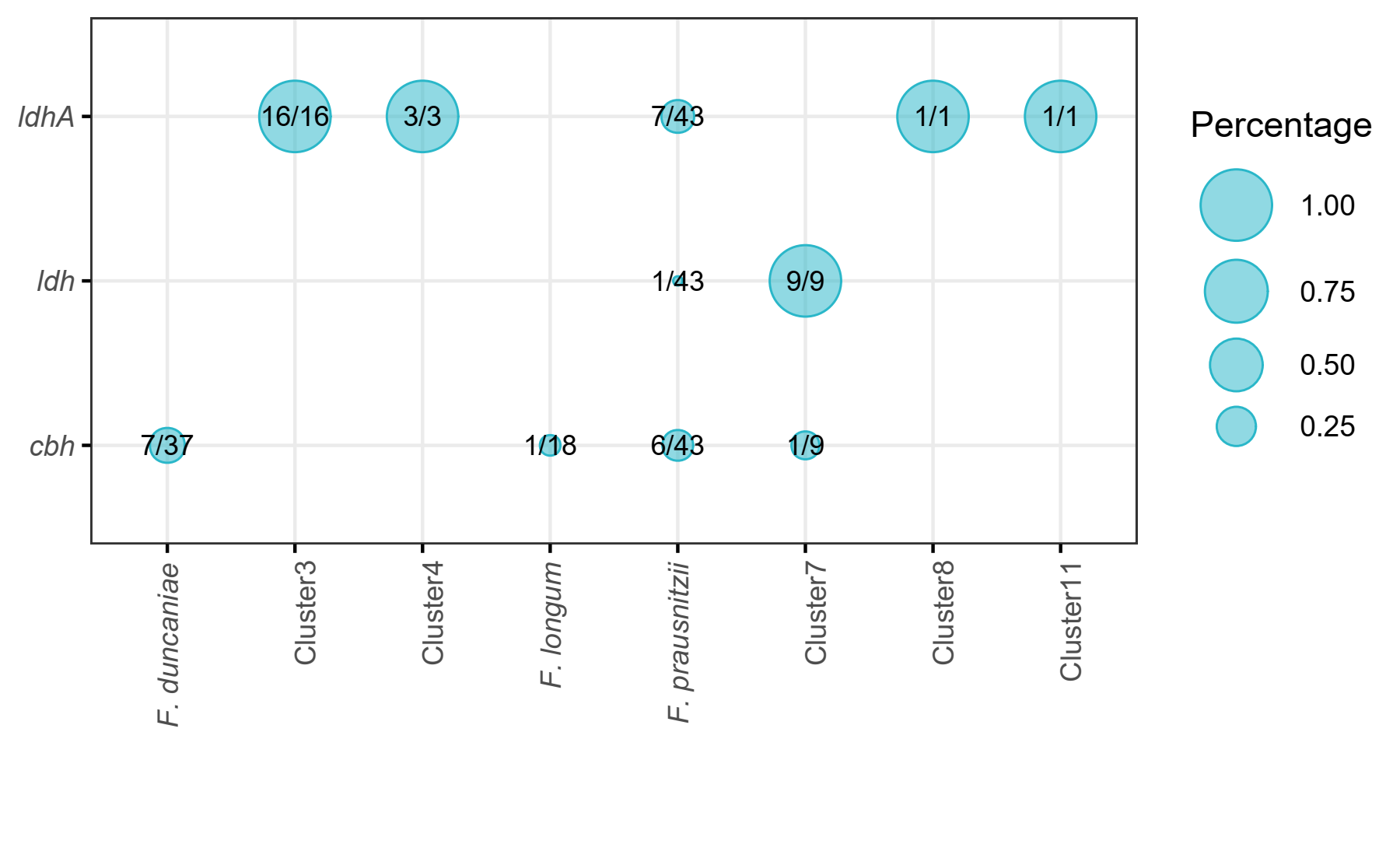


**Figure S5. The other probiotic function profile of *Faecalibacterium*.** The bubble plot shows the genes encoding L-lactate dehydrogenase (*ldh*), D-lactate dehydrogenase (*ldhA*) and bile salt hydrolase (*cbh*). The number in the circle represents the number of genomes harboring the corresponding gene in the total number of genomes of the species. The size of the circle represents the percentage of the number of genomes harboring the corresponding gene in the total number of genomes of the species.


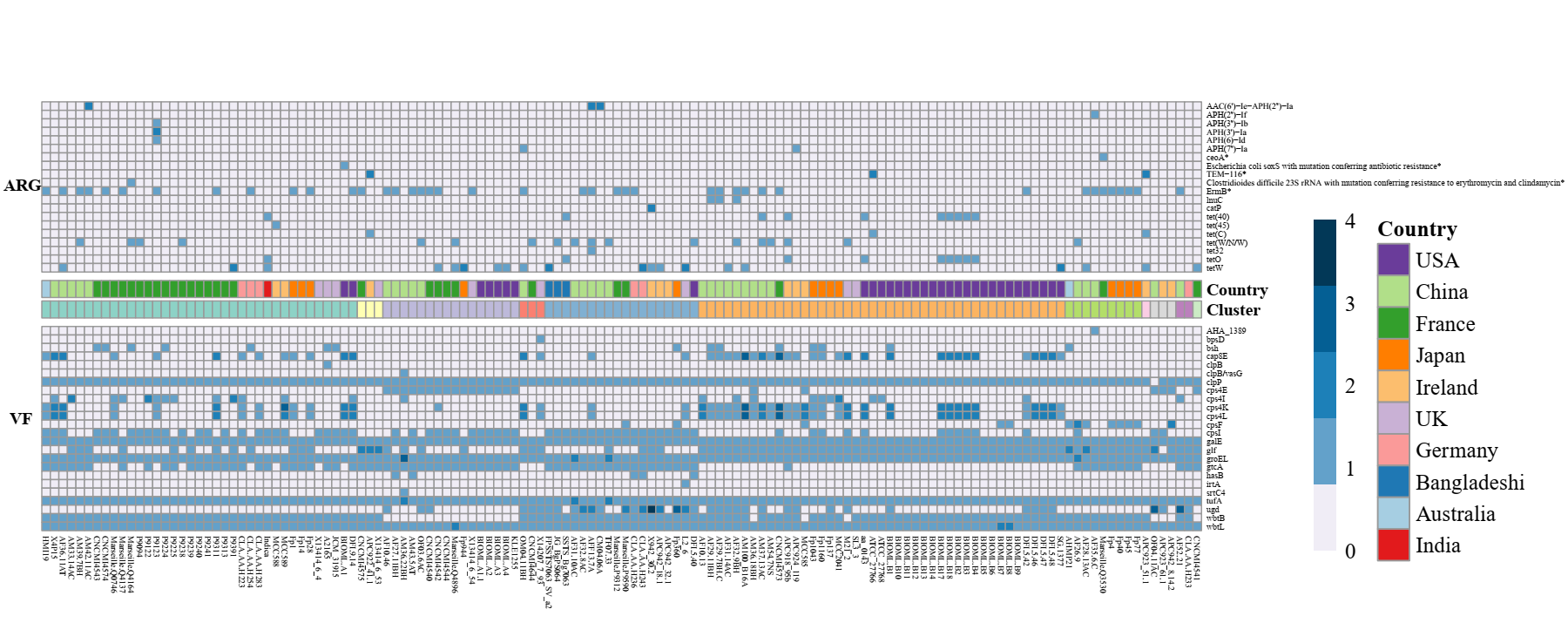
 **Figure S6. The safety evaluation profile of *Faecalibacterium*.** The heatmap shows the number of ARGs (above) and virulence factors (below) harbored by the genomes, colored according to the clusters and the countries.
